# Cardiorespiratory impact of intrathoracic pressure overshoot during artificial carbon dioxide pneumothorax: a randomized controlled study

**DOI:** 10.1186/s12871-022-01621-9

**Published:** 2022-03-23

**Authors:** Yunqin Ren, Xing Zhu, Hong Yan, Liyong Chen, Qingxiang Mao

**Affiliations:** Department of Anesthesiology, Daping Hospital, Army Medical University, 10 ChangjiangZhilu, Yuzhong District, 400042 Chongqing, China

**Keywords:** Capnothorax, Insufflator, Intrathoracic pressure, Overshoot, Thoracoscopy

## Abstract

**Background:**

The aim of this study is to evaluate cardiovascular and respiratory effects of intrathoracic pressure overshoot (higher than insufflation pressure) in patients who underwent thoracoscopic esophagectomy procedures with carbon dioxide (CO_2_) pneumothorax.

**Methods:**

This prospective research included 200 patients who were scheduled for esophagectomy from August 2016 to July 2020. The patients were randomly divided into the Stryker insufflator (STR) group and the Storz insufflator (STO) group. We recorded the changes of intrathoracic pressure, peak airway pressure, blood pressure, heart rate and central venous pressure (CVP) during artificial pneumothorax. The differences in blood gas analysis, the administration of vasopressors and the recovery time were compared between the two groups.

**Results:**

We found that during the artificial pneumothorax, intrathoracic pressure overshoot occurred in both the STR group (8.9 mmHg, 38 times per hour) and the STO group (9.8 mmHg, 32 times per hour). The recorded maximum intrathoracic pressures were up to 58 mmHg in the STR group and 51 mmHg in the STO group. The average duration of intrathoracic pressure overshoot was significantly longer in the STR group (5.3 ± 0.86 s) vs. the STO group (1.2 ± 0.31 s, *P* < 0.01). During intrathoracic pressure overshoot, a greater reduction in systolic blood pressure (SBP) (5.6 mmHg vs. 1.1 mmHg, *P* < 0.01), a higher elevation in airway peak pressure (4.8 ± 1.17 cmH_2_O vs. 0.9 ± 0.41 cmH_2_O, *P* < 0.01), and a larger increase in CVP (8.2 ± 2.86 cmH_2_O vs. 4.9 ± 2.35 cmH_2_O, *P* < 0.01) were observed in the STR group than in the STO group. Vasopressors were also applied more frequently in the STR group than in the STO group (68% vs. 43%, *P* < 0.01). The reduction of SBP caused by thoracic pressure overshoot was significantly correlated with the duration of overshoot (*R* = 0.76). No obvious correlation was found between the SBP reduction and the maximum pressure overshoot.

**Conclusions:**

Intrathoracic pressure overshoot can occur during thoracoscopic surgery with artificial CO_2_ pneumothorax and may lead to cardiovascular adverse effects which highly depends on the duration of the pressure overshoot.

**Trial registration:**

Clinicaltrials.gov (NCT02330536; December 24, 2014).

**Supplementary Information:**

The online version contains supplementary material available at 10.1186/s12871-022-01621-9.

## Background

Video-assisted thoracoscopic surgery (VATS) has been widely used in the diagnosis and treatment of a variety of thoracic diseases. To facilitate endoscopic visualization of intrathoracic structures, collapse of the ipsilateral lung is required. One-lung ventilation though double-lumen endotracheal tube (DLET) intubation, provides excellent intrathoracic exposure. However, DLET intubation can lead to a higher incidence of tracheobronchial injury, a longer time of DLET placement and an increased cost for patients [1, 2]. Besides, DLET intubation is not suitable for pediatrics or patients with difficult airways. Two-lung ventilation with CO_2_ insufflated into unilateral thoracic cavity can also afforded an excellent working space to perform many thoracoscopic surgeries including sympathectomies, wedge resections and esophagectomies [1, 3, 4]. However, insufflation of CO_2_ creates physiologic effects similar to a tension pneumothorax. Acute changes of intrathoracic pressure during the initial insufflation of CO_2_ can compromise cardiopulmonary function and patient safety [5–7]. Currently, few reports have observed the characteristics of intrathoracic pressure after artificial CO_2_ pneumothorax has been established and its potential impacts on cardiorespiratory function. During laparoscopy, Jacobs found that the real intraabdominal pressure peaks temporarily reached up to 1.8 times of the pre-set value, namely pressure overshoot [8].

Therefore, we conducted the present study to observe the real intrathoracic pressure during artificial pneumothorax and compared the hemodynamic and respiratory effects of intrathoracic pressure overshoot in patients who underwent thoracoscopic esophagectomy with two different insufflators.

## Methods

### Study design and ethics

 This study was approved by the ethics committee of Daping Hospital, Army Medical University (IRB 2014-9) and written informed consent was obtained from all subjects participating in the trial. The trial was registered prior to patient enrollment at clinicaltrials.gov (NCT02330536, Principal investigator: Qingxiang Mao, Date of registration: December 24, 2014). All patients were randomly allocated to two groups, Stryker insufflator (STR) group and Storz insufflator (STO) group, by a computerized randomization table. This article adheres to the applicable Consolidated Standards of Reporting Trials (CONSORT) guidelines.

### Patient population

From August 2016 to July 2020, a total of 228 patients who were scheduled for elective thoracoscopic esophagectomies with artificial pneumothorax were enrolled (Figure S1). Inclusion criteria included a definite diagnosis of esophageal cancer and a willingness to be treated by thoracoscopy combined with carbon dioxide artificial pneumothorax. Exclusion criteria included: age > 80 years, severe cardiovascular or respiratory diseases, American Society of Anesthesiologist (ASA) Grade > 3 and being unable or unwilling to give written consent to this clinical study. Patients who were converted to thoracotomy (including 3 cases with unexpected massive hemorrhage and 5 cases with severe thoracic adhesion), had significant intraoperative blood loss (> 400ml) or sustained severe arrhythmia during the operation were also excluded from the study. This study finally included 172 males and 28 females with a mean age of 66.75 years (Table 1). All of the surgeries were performed by the same team in the Department of Thoracic Surgery, Daping Hospital, Army Medical University (Chongqing, China).

**Table 1:** Baseline characteristics of participating patients, values are mean (SD) or number (proportion)

Characteristics	STR Group (*n* = 100)	STO Group (*n* = 100)	*P*
Height (cm)	162 ± 6.6	165 ± 5.6	0.412
Weight (kg)	63 ± 9.7	60 ± 7.4	0.476
Age (year)	67 ± 7.6	67 ± 3.1	0.864
Sex (male/female)	88 / 12	84 / 16	0.870
Operation time (min)	235 ± 32.6	259 ± 66.7	0.140
Pneumothorax time (min)	78 ± 19.8	90 ± 31.3	0.128
^a^Anesthesia recovery time (min)	38.5 ± 5.24	36.2 ± 5.45	0.152
Vasopressor usage rate (%)	68%	43%	0.001
Intraoperative blood loss (ml)	122 ± 45.9	118 ± 55.8	0.790
Fluid replacement volume (ml)	2182 ± 503.7	2290 ± 455.4	0.489
Urine volume (ml)	454 ± 241.1	453 ± 206.2	0.991

^a^ Anesthesia recovery time: the time from the end of anesthesia till the patient returned to the ward

### Anesthesia management

At admission to the operating room, the patients were monitored with electrocardiogram (ECG), oxygen saturation (SpO_2_), non-invasive blood pressure (NBP) and bispectral index (BIS). Anesthesia was induced with midazolam (30 to 40 µg.kg^−1^), sufentanil (0.5 µg.kg^−1^), propofol (1.5 to 2.0 mg.kg^−1^), and cisatracurium (0.2 mg.kg^−1^). Trachea was intubated with a single-lumen endotracheal tube. Anesthesia was maintained with inhaled sevoflurane and continuous infusion of remifentanil and propofol to maintain the BIS value within 40-60. Catherization of the right internal jugular vein and the left radial artery were performed to monitor central venous pressure (CVP) and arterial blood pressure (ABP), respectively.

Patients were infused with 6 ml.kg^−1^ of Ringer’s lactate solution before anesthesia induction and infused with 6-8 ml.kg^−1^.hour^−1^ fluid including crystalloid and colloid (2:1 ratio of crystalloid to colloid) during the operation. If intraoperative systolic blood pressure (SBP) was lower than 20% of the baseline or less than 90 mmHg (hypotension) and lasted more than 3 min, norepinephrine (0.01-0.1 µg.kg^−1^.min^−1^) would be administrated to maintain the blood pressure over the hypotension threshold.

Mechanical ventilation with 100% oxygen was initiated with a tidal volume of 7 ml.kg^−1^ and respiratory rate of 13 breaths.min^−1^ (non-artificial pneumothorax period). During artificial pneumothorax, the tidal volume was 5ml.kg^−1^ and the respiratory rate was 20 breaths.min^−1^, and the inspiratory: expiratory ratio was maintained at 1:2.

### Induction and maintenance of artificial pneumothorax

Patients were placed in a 30° left-lateral tilt position. After a disposable trocar was inserted into the pleural cavity, the CO_2_ was insufflated into the thoracic cavity via an insufflator. The insufflation pressure and the flow rate were set as 8 mmHg and 20 l.min^−1^. An invasive blood pressure transducer filled with saline was connected to another trocar and used to monitor the real-time intrathoracic pressure. The patients were randomly divided into two groups: the STR group, in which artificial pneumothorax was performed by Stryker insufflator F102 (Stryker corporation, Kalamazoo, MI, USA); the STO group, in which artificial pneumothorax was performed by Storz insufflator 0U32540 (Karl Storz GmbH & Co, Tuttlingen, Germany).

### Surgical procedure of VATS

**Fig. 1:**
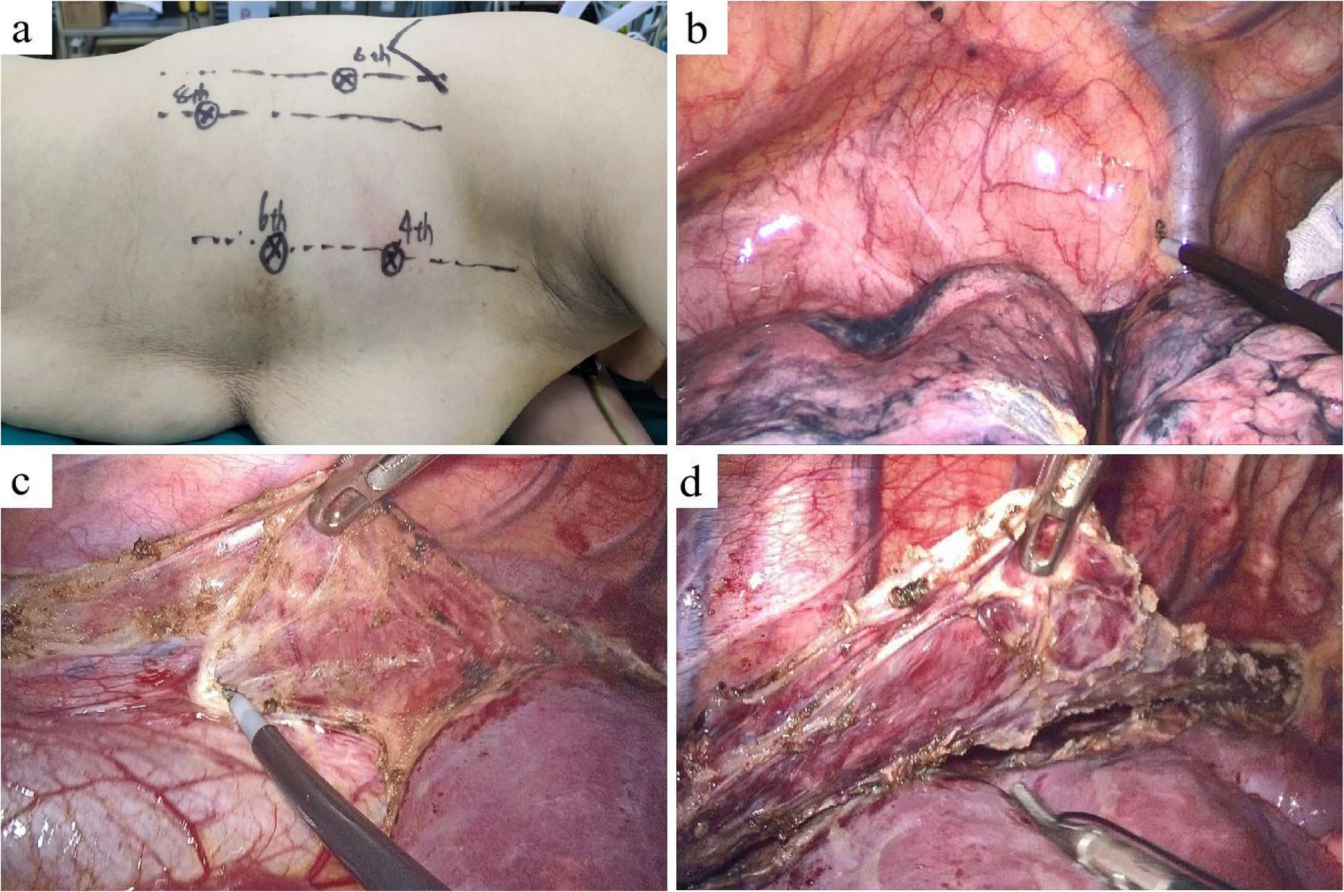
Thoracoscopic procedures of thoracoscopic-laparoscopic esophagectomy. **a** Patients were placed in the semi-prone position and four trocars were placed at four sites. **b**-**d** Intraoperative images during artificial pneumothorax

Briefly, patients were intubated with a single-lumen endotracheal tube and placed in the semi-prone position. Four trocars were placed at four sites, including the camera trocar (12 mm) in the sixth intercostal space on the anterior axillary line, one 5-mm trocar in the sixth intercostal space on the anterior axillary line, one 5-mm trocar in the sixth intercostal space on the posterior axillary line, and one 12-mm trocar in the eighth intercostal space on the posterior axillary line (Fig. 1a). After artificial pneumothorax was performed, the esophagus was mobilized caudally to the esophageal hiatus and cranially to the thoracic entrance, accompanied by lymphadenectomy of supraphrenic, paraesophageal, peripulmonary vein, subcarinal, right and left recurrent laryngeal nerve, and paratracheal stations (Fig. 1b and d).

### Data collection

A video camera was used to record the changes in intrathoracic pressure, ABP, ECG, SpO_2_, CVP, and airway pressure of the patients in two groups during artificial pneumothorax. The arterial blood gas was measured at 5 min before artificial pneumothorax started (T1) and 5 min after artificial pneumothorax stopped (T2). The usage of vasopressors and the postoperative recovery time were also recorded.

### Statistical analysis

Numerical data were reported as mean ± standard deviation and was statistically analyzed using 2-sided *t* test or two-way ANOVA followed by the *post hoc* Turkey method. Count data were analyzed with the Chi-square test. Multiple linear regression analysis was conducted for the correlation analysis between pressure overshoot and cardiopulmonary function changes. A value of *P* < 0.05 was considered statistically significant. SPSS 19.0 software (SPSS Inc., Chicago, IL, USA) was used for statistical analysis. Almost 200 patients per group were required to detect a 15% difference in the pressure overshoot value between two group (alpha = 0.01, power = 0.9). The level of significance was set at *P* < 0.01.

## Results

### Patient characteristics

No significant difference was found in the height, weight, age, and gender between the STR group and the STO group (*P* > 0.05). In addition, the clinical parameters including operation time, pneumothorax time, and anesthesia recovery time were similar between the two groups (*P* > 0.05). The vasopressor usage rate was significant higher in STR group than in STO group (STR: 68%, STO: 43%, *P* = 0.001). However, there was no significant difference between the two groups in intraoperative blood loss (STR: 122 ± 45.9 ml, STO: 118 ± 55.8 ml, *P* = 0.790), fluid replacement volume (STR: 2182 ± 503.7 ml, STO: 2290 ± 455.4 ml, *P* = 0.489) and urine volume (STR: 454 ± 241.1 ml, STO: 453 ± 206.2 ml, *P* = 0.991) (Table 1).

### The profiles of thoracic pressure overshoot and its impact on the circulatory and respiratory functions

**Fig. 2:**
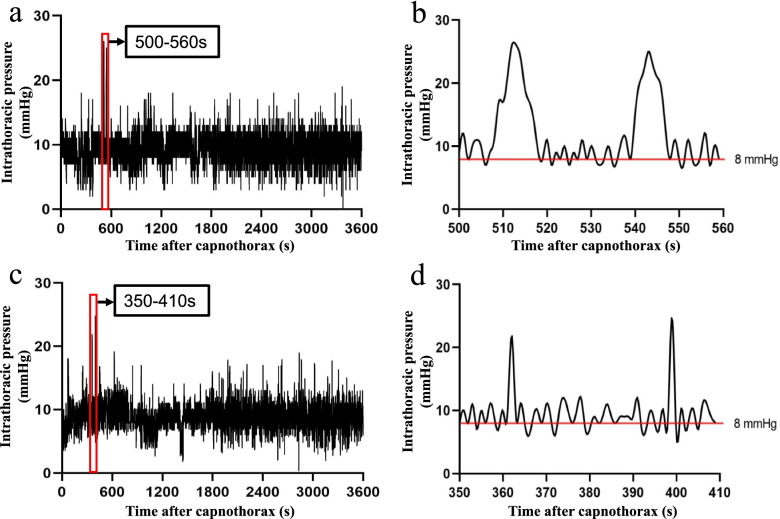
Examples for intrathoracic pressure changes in the STR and the STO group. **a** Typical graph of intrathoracic pressure changes in the STR group during artificial pneumothorax within 60 min. **b** A detailed view of the changes in intrathoracic pressure during the 500 – 560 s in a. **c** Typical graph of intrathoracic pressure changes in the STO group during artificial pneumothorax within 60 min. **d** A detailed view of the changes in intrathoracic pressure during the 350 – 410 s in c

Constant fluctuations in intrathoracic pressure of the two groups were observed during the entire artificial pneumothorax (Fig. 2a and d). One possible reason was that the surgical suction and gas leakage from the trocar puncture would decrease the intrathoracic pressure and re-initiate the CO_2_ insufflation. In addition to CO_2_ insufflation, mechanical ventilation can also cause periodic fluctuations in intrathoracic pressure of the pneumothorax side. However, this increased intrathoracic pressure induced by the mechanical ventilation was generally less than 3 mmHg (Fig. 2b and d). Thus, in this study, the threshold of intrathoracic pressure overshoot events caused by CO_2_ insufflation was defined as an increased pressure that was greater than 3 mmHg. We found that pressure overshoot occurred in both of the two groups during the artificial pneumothorax, and there was no significant difference in the frequency (STR: 38 ± 7.4 times.hour^−1^; STO: 32 ± 9.9 times.hour^−1^, *P* = 0.238) and the magnitude (STR: 8.9 ± 1.60 mmHg; STO: 9.8 ± 3.13 mmHg, *P* = 0.551) of pressure overshoot (defined as the maximum thoracic pressure minus eight mmHg) between the two groups. However, a longer pressure overshoot duration (5.3 ± 0.86 s vs. 1.2 ± 0.31s, *P* < 0.01) as well as a larger increase in peak airway pressure (4.8 cmH_2_O vs. 0.9 cmH_2_O, *P* < 0.01), a greater rise in CVP (8.2 cmH_2_O vs. 4.9 cmH_2_O, *P* < 0.01), a greater decrease in SBP (5.6 mmHg vs. 1.1 mmHg, *P* < 0.01), and a higher percentage of patients with significant SBP drop (drop > 10 mmHg) were found in the STR group than those in the STO group. No significant difference was observed in the magnitude of heart rate reduction (0 ± 1.4 bpm vs. 0 ± 1.3 bpm, *P* = 0.534) and the percentage of patients with SpO_2_ reduction (0% vs. 0%, *P* = 1.000) between the two groups (Table 2).

**Table 2:** The profiles of intrathoracic pressure overshoot and its impact on the circulatory and respiratory functions

	STR group (*n* = 100)	STO group (*n* = 100)	*P*
Pressure overshoot frequency (times.hour^−1^)	38 ± 7.4	32 ± 9.9	0.238
Pressure overshoot duration (second)	5.3 ± 0.86	1.2 ± 0.31	0.000
Pressure overshoot value (mmHg)^a^	8.9 ± 1.60	9.8 ± 3.13	0.551
Maximum pressure overshoot (mmHg)	58	51	NULL
Increase of peak airway pressure (cmH_2_O)^b^	4.8 ± 1.17	0.9 ± 0.41	0.000
Increase of central venous pressure (cmH_2_O)^c^	8.2 ± 2.86	4.9 ± 2.35	0.000
Decrease in SBP (mmHg)	5.6 ± 1.47	1.1 ± 0.39	0.000
Percentage of patients with significant SBP drop^d^	51%	9%	0.000
Decrease in heart rate (beats per minute)	0 ± 1.4	0 ± 1.3	0.534
SpO_2_ reduction percentage	0%	0%	1.000

^a^Pressure overshoot value: defined as the maximum thoracic pressure minus eight mmHg during each pressure overshoot period

^b^Increase of peak airway pressure (cmH_2_O): defined as the maximum peak airway pressure in each pressure overshoot minus the baseline in the pre-overshoot period

^c^Increase of central venous pressure: defined as the maximum CVP during each pressure overshoot minus the baseline in the pre-overshoot period

^d^Percentage of patients with significant SBP drop: defined as the percentage of patients who suffered at least once the SBP drop magnitude was more than 10 mmHg during pressure overshoot

### Cardiovascular inhibition caused by the thoracic pressure overshoot was highly correlated with the duration of overshoot

**Fig. 3:**
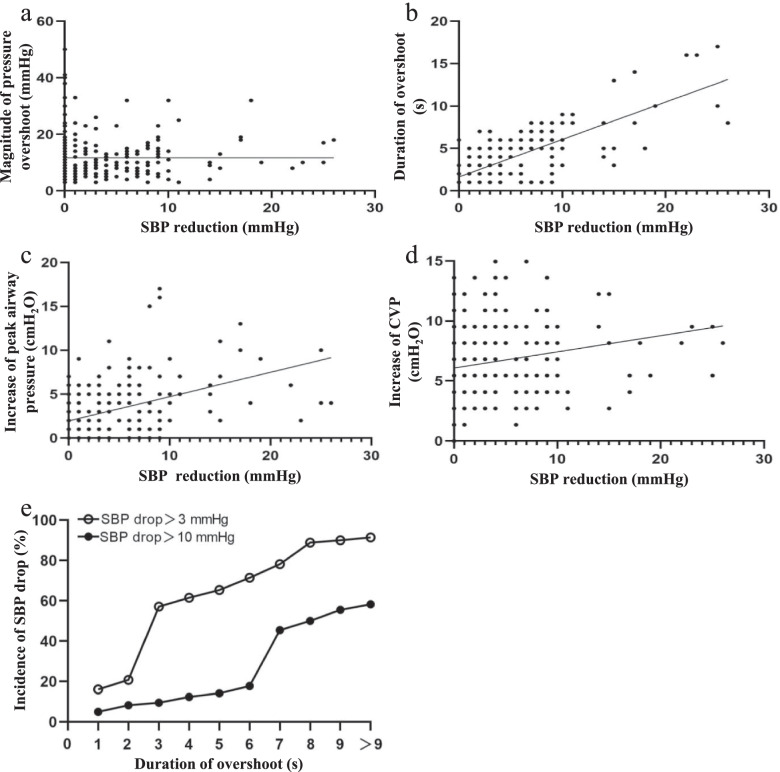
The correlation between the SBP reduction and the magnitude of pressure overshoot, duration of overshoot, increase of peak airway pressure and increase of CVP, respectively. **a** Scatter plot of the magnitude of pressure overshoot and the SBP reduction. **b** Scatter plot of the duration of overshoot and the SBP reduction. **c** Scatter plot of the increase in peak airway pressure and the SBP reduction. **d** Scatter plot of the increase in CVP and the SBP reduction. **e** Trend graph showed the relationship between the duration of pressure overshoot and the incidence of SBP drop (defined as SBP drop was more than 5 mmHg during pressure overshoot)

Since the higher incidence and magnitude of SBP drop and a longer overshoot duration were found in the STR group, we assumed that whether the cardiovascular inhibition caused by the thoracic pressure overshoot depended on the overshoot duration. To address this question, we pooled the data from the STR and STO group together and performed the multiple linear regression analysis for the correlation between the magnitude of thoracic pressure overshoot, the duration of pressure overshoot, the increase of peak airway pressure, the increase of CVP and the SBP drop, respectively. The analysis indicated that there was no significant correlation between the SBP reduction and the intrathoracic pressure overshoot (*R* = 0.00, *P* = 0.996) (Fig. 3a) while a strong correlation was found between the decrease in blood pressure and the duration of intrathoracic pressure overshoot (*R* = 0.76, *P* < 0.01) (Fig. 3b). Additionally, the decrease in blood pressure and the change in peak airway pressure were moderately correlated (*R* = 0.47, *P* < 0.01) (Fig. 3c). A weak correlation was also detected between the decrease in blood pressure and the increase in central venous pressure (*R* = 0.22, *P* < 0.01) (Fig. 3d).

To investigate the effect of duration of pressure overshoot on the incidence of SBP drop with different magnitudes (> 3 mmHg, > 10 mmHg), the duration of intrathoracic pressure overshoot was divided by one second interval and into 10 levels as below: ≤ 1 s, 1-2 s, 2-3 s, 3-4 s, 4-5 s, 5-6 s, 6-7 s, 7-8 s, 8-9 s and > 9 s. The incidences of SBP drop with different magnitudes all increased with the overshooting time. When the overshoot time exceeded 2 s, the incidence of more than 3 mmHg SBP drop rapidly increased to 57.14%, and when the overshoot time exceeded 6 s, the incidence of more than 10 mmHg SBP drop rapidly increased to 45.45%, which indicated that there was a threshold effect of overshoot time on the SBP drop (Fig. 3e).

### Changes of blood gas analysis after artificial pneumothorax

To investigate the effect of artificial pneumothorax on the respiratory function, we compared the blood gas analysis results between the STR group and the STO group at 5 min before the artificial pneumothorax (T1) and 5 min after the artificial pneumothorax stopped (T2). The results showed that arterial partial pressure of CO_2_ (PaCO_2_) significantly increased (*P* < 0.01) while the pH value (*P* < 0.01) and base excess (BE) significantly decreased (*P* < 0.01) at T2 than those at T1 in both groups. No significant difference was found in blood lactic acid (Lac) and arterial partial pressure of oxygen (PaO_2_) between T1 and T2 in both groups. There were no statistically differences of blood gas analysis between two groups at either T1 or T2 (*P* > 0.05) (Table 3).

**Table 3:** Comparison of blood gas analysis before and after artificial pneumothorax between the STR and the STO groups

Group	Time	pH	PaO_2_(mmHg)	PaCO_2_(mmHg)	BE(mmol.l^−1^)	Lac(mmol.l^−1^)
STR	T1	7.41±0.05	372.8±54.42	39.20±5.99	0.28±2.50	0.89±0.26
	T2	7.32±0.06^*^	335.2±58.05	47.64±6.37^*^	-1.70±2.20^*^	0.83±0.19
STO	T1	7.39±0.05	359.4±43.72	42.31±4.76	0.21±2.22	0.94±0.40
	T2	7.32±0.07^#^	329.6±45.03	47.69±5.82^#^	-1.70±2.37^#^	0.82±0.16

*: T1 vs. T2 in STR group, *P* < 0.05; #: T1 vs. T2 in STO group, *P* < 0.05

## Discussion

CO_2_ artificial pneumothorax has been widely used in cardiothoracic surgery, which not only helps surgeons obtain the optimal visualization of surgical field and operation space, but also makes it possible to perform thoracoscopic surgery for patients who are not suitable for intubation with DLET(such as children or patients with difficult airway) [9, 10]. However, CO_2_ artificial pneumothorax may also have several limitations, including the occurrence of intrathoracic pressure overshoot. Although the insufflation pressure of CO_2_ is usually fixed, the real thoracic pressure is not presumably constant due to the need for surgical suction and gas leakage from the trocar puncture. The insufflator will insufflate CO_2_ into the chest cavity again to reach the pre-set pressure. The insufflation volume may exceed the actual required volume because of the delayed response between the pressure monitoring system and the flow control regulator [11]. Then the intrathoracic pressure will be higher than the pre-set value, that is, the pressure overshoot that occurs. Modern insufflators have been adopted by several ways to reduce the magnitude and frequency of pressure overshoot, including constant pressure and variable flow mode (namely, the closer the insufflation pressure is to the pre-set value, the lower the flow rate is), active pressure relief, etc. However, intrathoracic pressure overshoot still cannot be completely eliminated. Jacobs et al. reported that the intra-abdominal pressure is not constant during CO_2_ pneumoperitoneum, and the pressure overshoot reached up to 1.8 times of the pre-set value [8]. In this study, we also found that intrathoracic pressure is not constant during CO_2_ artificial pneumothorax, which was consistent with previous reports [8, 11, 12]. We also found that the intrathoracic pressure overshot value can reach up to 7 times of the pre-set value.

Intrathoracic pressure overshoot can impair the cardiovascular function, which is mainly due to the compresses on the heart and large blood vessels, leading to the obstructions in venous return and the inhibition of cardiac systolic and diastolic functions [13, 14]. The severity of cardiovascular instability depends on the pneumothorax pressure, the insufflation flowrate, and the individual compensatory to pneumothorax [15]. Currently, CO_2_ pneumothorax with insufflation pressure of 8 mmHg is widely accepted as the following cardiovascular compromise is less obvious [16, 17]. In previous reported cardiovascular collapse events during CO_2_ insufflation, the insufflation pressure was higher than 8 mmHg which implied a potential higher intrathoracic pressure overshoot and a more severe cardiovascular comprise [5, 6]. In present study, the CO_2_ insufflation pressure was set at 8 mmHg. However, we still found that pressure overshoot during artificial pneumothorax could compromise cardiovascular function as evidenced by the significant SBP drop (>10 mmHg) events affected 51% patients in the STR group and 9% patients in the STO group.

Interestingly, we found that the frequency and magnitude of pressure overshoot in the STO group were not significantly different from those in the STR group except for the shorter duration of pressure overshoot (1.2 s vs. 5.3 s). The pressure overshoot in the STO group appeared to have less effect on cardiopulmonary function. The percentage of vasopressors usage, the increase in peak airway pressure, the increase in central venous pressure and the decrease in blood pressure during artificial pneumothorax in the STO group were all less than those in the STR group. Then a correlation regression analysis was performed and found that SBP drop was highly correlated with the pressure overshoot duration (R = 0.76) while not the magnitude of pressure overshoot. The longer the pressure overshoot lasts, the higher the risk of blood pressure reduction goes. We also found that there was a threshold effect of overshoot duration time on the SBP drop which is that overshoot duration longer than 2 or 6 s will lead to a significant increased incidence of SBP drop > 3 mmHg or incidence of SBP drop > 10 mmHg, respectively. These results imply that different brands of insufflators may have different characteristics of pressure overshoot due to discrepancies in electrical mechanism, which may exert distinctive effects on the cardiovascular function. Besides, a significant correlation was also detected between SBP reduction and peak airway pressure increase. One possible explanation may be since the peak airway pressure always occurred in the inspiratory phase, and the inspiration of lung would reduce the intrathoracic space and further increase the pressure overshoot. Then the further increased pressure overshoot may lead to SAP reduction. Whether the increase in peak airway pressure is the direct cause of the decrease in SBP still needs to be confirmed by further experiments.

CO_2_ pneumothorax can lead to the increased airway pressure, the decreased oxygenation, and the hypercapnia [18]. In addition to the impact of artificial pneumothorax, intrathoracic pressure overshoot can further increase the peak inspiratory pressure which may cause more stress damage to the pulmonary alveoli [19, 20]. However, in our study there was no significant decrease in the oxygenation index of the STR group when compared with the STO group. This could be because that the duration and magnitude of the increased peak inspiratory pressure of the STR group are not long and high enough to exert significant alveolar damage. Both the STR group and the STO group had obvious hypercapnia and acidosis after artificial pneumothorax, but there was no significant difference between the two groups, indicating that the difference of pressure overshoot between those two groups have little effect on the absorption and discharge of CO_2_.

This study has several limitations. First, this is a single-center randomized controlled study and we excluded patients who were converted to thoracotomy, had massive intraoperative blood loss or sustained severe arrhythmia during the operation, the potential selection bias may limit generalization of our results to other populations. Second, we only analyzed the intraoperative circulatory and respiratory functions during intrathoracic pressure overshoot, the potential damage of pressure overshoot to other organs and the long-term outcomes of patients were not studied. Another potential effect of capnothorax is that intraoperative venous bleeding may be covered when intrathoracic pressure exceeds venous pressure and the negative intrathoracic pressure during spontaneous inspiration could siphon blood into the pleural cavity [21]. This potential risk for patient’s outcome needs further evaluation. Third, we only compared the overshoot characteristics of two types of insufflators. A further work is needed to clarify the potential effects of other types of insufflators on the intrathoracic pressure. Last, all the patients in this study are adults, whether the pressure overshoot has a different cardiopulmonary impact on children patients needs to be further explored.

In conclusion, during CO_2_ artificial pneumothorax, intrathoracic pressure overshoot can occur and lead to cardiovascular compromise. The longer the pressure overshoot lasts, the higher the risk of blood pressure reduction goes.

## Supplementary Information

**Additional file 1: Figure S1.** Patient screening and exclusion process. Total 228 patients participated in the study and 28 patients were excluded.

## Data Availability

All data generated or analysed during this study are included in this published article.

## Abbreviations

ABP: arterial blood pressure
ASA: American Society of Anesthesiologist
BIS: bispectral index
CO_2_: carbon dioxide
CVP: central venous pressure
DLET: double-lumen endotracheal tube
ECG: electrocardiogram
NBP: non-invasive blood pressure
SBP: systolic blood pressure
SpO_2_: oxygen saturation
STO: Storz insufflator
STR: Stryker insufflator
VATS: Video-assisted thoracoscopic surgery
