# Supplementary figures and images for: Cardiorespiratory impact of intrathoracic pressure overshoot during artificial carbon dioxide pneumothorax: a randomized controlled study
Source: BMC Anesthesiol. 2022 Mar 23;22:76. doi: 10.1186/s12871-022-01621-9 (PMC8941761; doi:10.1186/s12871-022-01621-9)

The CONSORT flow diagram of the human study

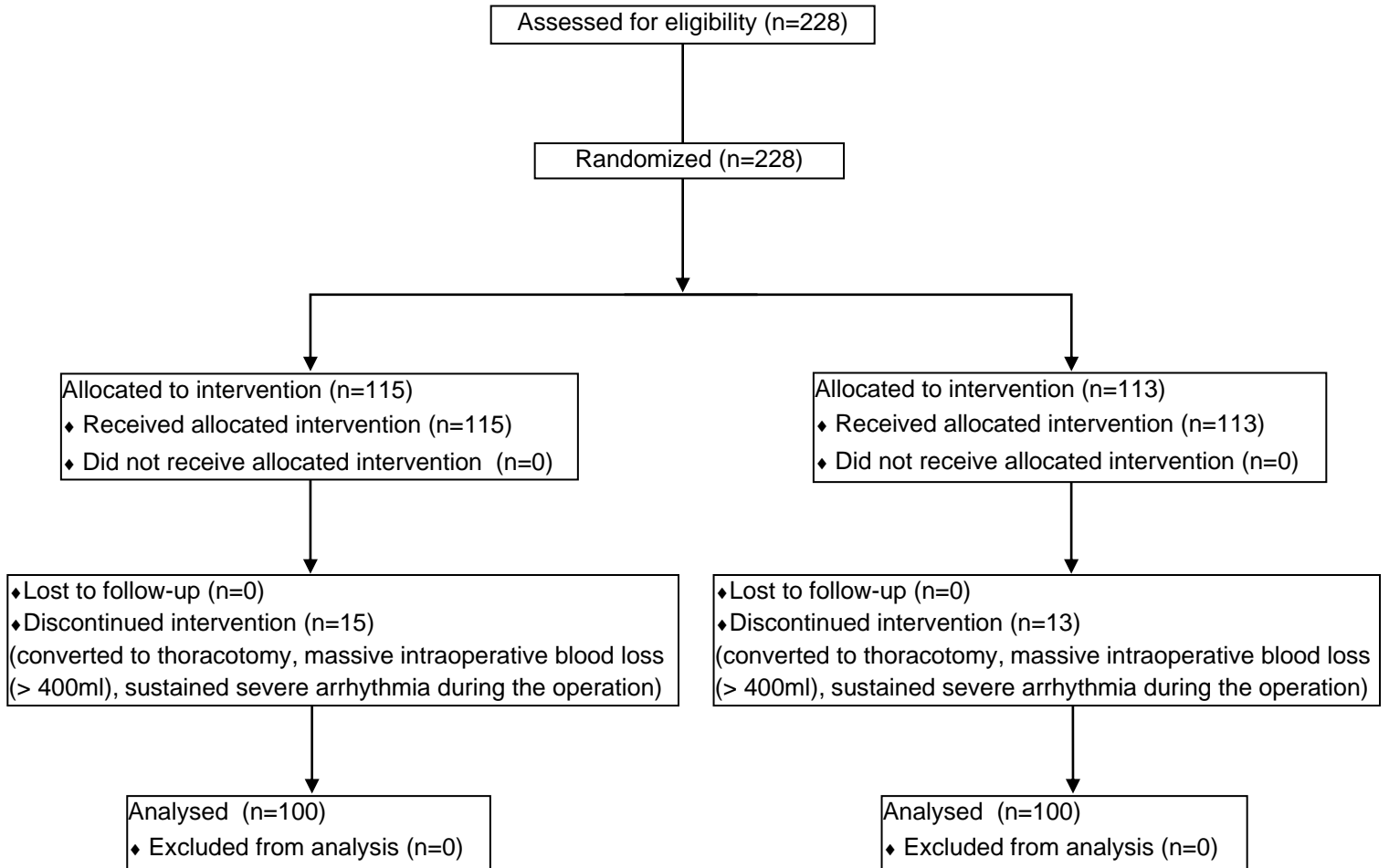

Supplement: Supplementary file 1 — Additional file 1: Figure S1. Patient screening and exclusion process. Total 228 patients participated in the study and 28 patients were excluded. [file 12871_2022_1621_MOESM1_ESM.pdf]
